# Drought does not induce crassulacean acid metabolism (CAM) but regulates photosynthesis and enhances nutritional quality of *Mesembryanthemum crystallinum*

**DOI:** 10.1371/journal.pone.0229897

**Published:** 2020-03-06

**Authors:** Jie He, Ee Lyn Chua, Lin Qin

**Affiliations:** National Institute of Education, Nanyang Technological University, Singapore, Singapore; National University of Kaohsiung, TAIWAN

## Abstract

Physiology and nutritional quality of a facultative CAM plant *Mesembryanthemum crystallinum* under drought stress alone are poorly understood. To induce drought, *M*. *crystallinum* was cultured aeroponically with different nutrient spraying intervals such as 5, 30, 60 and 240 min. The long spraying interval such as 240 min resulted in lower mass of root and shoot, shorter total root length with less tips and smaller surface area, compared to short interval of 5 min. Grown under the longest spraying interval of 240 min, *M*. *crystallinum*also had significantly higher leaf dry matter content but lower leaf succulence. However, *CAM acidity was undetectable for any plants*. Although *M*. *crystallinum* grown under extended spraying intervals had higher photosynthetic pigments, they utilized lesser light energy and did not dissipate heat as effectively as those grown under 5 min. Compare to other shorter spraying intervals, photosynthetic gas exchange rates were significantly reduced under 240 min spraying interval, indicating signs of water deficit stress. Shoot nitrate, total reduced nitrogen, total soluble protein and Rubisco concentrations were similar for all plants. For phytochemicals and dietary minerals, plants grown under 240 min spraying interval had significantly higher values than the other plants. Therefore, drought does not result in the *induction* of *CAM* but regulates photosynthetic performance and enhances nutritional quality of *M*. *crystallinum*.

## Introduction

Many studies demonstrated that crassulacean acid metabolism (CAM) was elicited if a drought- and salt-tolerant species *Mesembryanthemum crystallinum* (the common ice plant) following drought coupled with salinity [1,2] and salinity stress [3]. Apart from *M*. *crystallinum*, facultative CAM has also been demonstrated in many other drought- and slat-tolerant species [4,5]. However, there is a substantial reversion to C_3_ photosynthesis in facultative CAM species following the removal of stress [5]. Since the initial report on the *facultative CAM* features of *M*. *crystallinum* in 1972 [1], this species has been used as a model plant to study the switch between CAM and C_3_ photosynthetic modes under drought and salinity stress [5–8]

Under native conditions, *M*. *crystallinum* is highly tolerant to drought and salinity [9–11]. Due to the capability of switching from C_3_ to CAM photosynthesis, it can complete its life cycle on the soil containing NaCl as high as 500 mM, which is equivalent to that in seawater [2]. Salinity stress induced CAM in *M*. *crystallinum* was usually companied the accumulation of osmolytes that was viewed as adaptation of plants to water deficit [12–14]. Proline accumulation is a well-known response to salt and drought stress [15]. Paul and Cockburn [12] reported that the irrigation of *M*. *crystallinum* plants with 400 mM NaCl induced CAM that was accompanied by the accumulation of proline and pinitol that was constituted 71% of the soluble carbohydrate. Kumari et al. [14] suggested that proline and its analog are the important metabolites involved in salt tolerance of halophytes. Other researches also reported that proline is accumulated in halophytes under conditions of high salinity [16]. Salinity and drought stress usually trigger oxidative stress, forming reactive oxygen species (ROS) and leading to cell membrane damage, and cell death [17]. Bose et al. [18] reported that proline has antioxidant functions. Halophytes are able to synthesize certain determined secondary metabolites termed as natural antioxidants under stressful conditions. Natural antioxidants that exhibit antioxidant activities are phenolics, carotenoids and vitamins in plants and the synthesis of these antioxidants is stimulated in responses to environment stress [19].

With its well-known nutritional quality and the medicinal uses [20,21], *M*. *crystallinum* has been grown as a vegetable in some European countries, Australia, New Zealand [22] and Asia [23,24]. Our recent study showed that *M*. *crystallinum* performed C_3_ photosynthesis when supplied with adequate water using indoor aeroponic farming systems under LED lighting in a cool room where temperature was maintained 26˚C/28˚C (day/night) [24]. Results of our study suggest that compared to red or blue-LED alone, appropriate combination of red- and blue-LED lighting (red:blue = 9:1) enhanced shoot and root biomass and shoot/root ratio. Photosynthetic performance of *M*. *crystallinum* measured by photosynthetic light use efficiency and photosynthetic gas exchange during light period were also higher under combination of red- and blue-LED compared to red-LED alone) [24].

While different technologies for the production of leafy vegetables have developed to improve yield, there is an increasing interest of consumers towards their quality [25]. *M*. *crystallinum* is nowadays cultivated as popular vegetable crop and its young shoots with their succulent, mellow, slightly salty and sour tasting leaves are delicious flavored salad greens. Recently, *this* halophyte crop has been reported to be produced indoor or in the greenhouse with soilless culture such as hydroponic or aeroponic farming systems [23,24]. It was reported that the CAM capacity of *M*. *crystallinum* can only enhanced by saline irrigation [26] or drought stress couple with salinity [27]. However, there is very little work done on the effects of drought stress on physiology such as the induction of CAM photosynthesis and the photosynthetic performance of *M*. *crystallinum* plants when they are grown with soilless cultivation. It was reported that antioxidants are constitutive of certain species and they can be increased or decreased under conditions of environmental stresses [27]. Hence, it also is necessary to evaluate the productivity together with the quality of *M*. *crystallinum* cultivated under salt-*stress free conditions*. Since CAM photosynthesis could also be induced under drought coupled with salinity in *M*. *crystallinum* [2,27], it would also be interesting to test if *drought stress* alone could induce CAM activity of *M*. *crystallinum* and change its nutritional quality.

This study aimed to investigate if drought stress alone induces CAM photosynthesis of facultative species, *M*. *crystallinum*. It also attempted to investigate the impacts of drought stress on growth, photosynthetic performance and quality of *M*. *crystallinum*. All plants were grown indoor under red- and blue-LED lighting (red:blue = 9:1) as previously reported by our team [24] with different nutrient spray intervals (5, 15, 60 and 240 min). Shoot and root productivity and leaf growth, root morphology, plant water relations measured by leaf succulence, leaf dry matter content and water content and the accumulation of osmolytes such as proline and soluble sugar were investigated. Photosynthetic gas exchange, CAM acidity, photosynthetic pigments and light use efficiency were also studied. Nitrogen metabolism measured by nitrate (NO_3_^-^), total reduced nitrogen, soluble protein and Rubisco protein, and dietary minerals, ascorbic acids, and phenolic compounds were also determined. The findings of this project help advance existing understanding of *M*. *crystallinum* plant physiology under different amount of water and nutrient supplies. It also helps the growers to enhance productivity and quality of *M*. *crystallinum* at low production cost through water and nutrient management.

## Materials and methods

### Plant materials and experimental designs

After germination, *M*. *crystallinum* seedlings were placed under a 16-h photoperiod of light and exposed to a photosynthetic photon flux density (PPFD) of 100 μmol m^-2^ s^-1^ provided by high-pressure sodium lamps for 4 weeks. Seedling were then transplanted to indoor aeroponic farming systems and grown under red- and blue-LED lighting in the ratio of 9 to 1 with maximal PPFD of 250 μmol m^-2^ s^-1^ for a 16-h photoperiod [24]. They were supplied with modified full strength Netherlands Standard Solution [28] with 2.0 ± 0.2 mS cm^-1^ conductivity and pH 6.0 ± 0.2. The modified full strength Netherlands Standard Solution had the following composition: N, 191.89 ppm; P, 33.29 ppm; K, 309.00 ppm; Ca, 210.29 ppm; Mg, 60.04 ppm; S, 125.45 ppm; Fe, 9.36 ppm; B, 0.105 ppm; Mn, 0.238 ppm; Zn, 0.014 ppm; Cu, 0.015ppm; Mo, 0.048 ppm; Na, 3.860 ppm. The *room temperature and relative humidity were 25°C/28°C (day/night)* and 60%/65% (day/night), respectively. All plants were acclimatized under the aforementioned conditions for three days with nutrient spraying intervals at 5 min before applying different nutrient spraying intervals at 5, 30, 60 and 240 min. The nutrient solution was pumped through discharge pipes installed at the base of the aeroponics trough. The duration of nutrient spraying was 1 min for all plants. The nutrient solution was programmed to spray at different intervals. This experiment was performed twice with similar results; results are presented from only one experiment. For each experiment, due to the large sample size, the tedious procedures and time-consuming for certain analysis, different measurements were carried on different days during the active growth stage, from 9 to 20 days after transplanting. Some parameters were measured twice on different days and similar results were obtained.

### Measurements of fresh weight (FW) and dry weight (DW) of root and shoot

After 15 days of transplanting, *M*. *crystallinum* plants under each nutrient spraying interval treatment were harvested. The polyurethane cube was removed from the roots and the shoots and roots were weighed separately to determine their FW. All samples were then wrapped individually in pre-weighed aluminium foil, dried at 80˚C for 4 days before measuring their DW.

### Measurements of leaf growth and leaf water status

After 15 days of transplanting, the total leaf number of *M*. *crystallinum* was first recorded. The leaf, stem and root were weighed separately to determine their FW. To obtain total leaf area, all the leaf margins were traced on A4 size paper and then weighed and compared to the weight of a known area of the same paper. Different plant tissues were then wrapped individually in pre-weighed aluminium foil, dried at 80˚C for 4 days before measuring their DW. The specific leaf area (SLA) was estimated as SLA = L_A_/L_DW_ with L_A_ = leaf area (cm^2^) and L_DW_ = leaf dry weight (g) according to Hunt et al. [29]. Leaf succulence = L_FW_/L_A_ with L_FW_ = leaf fresh weight (g) and L_A_, leaf area (cm^2^) [7]. According to Garnier et al. [30], leaf dry matter content (LDMC) and leaf water content (LWC) were estimated as LDMC = L_DW_/L_FW_ with L_DW_ = leaf dry weight (g) and L_FW_ = leaf fresh weight (g) and LWC = (L_FW_−L_DW_)/L_FW_.

### Root morphology analysis

After 9 days of transplanting, the root from each plant was detached and placed in a tray of water that served to spread the roots out. Using the WIN MAC RHIZO scanner, the roots were first scanned before using the WIN MAC RHIZO V 3.9 programme to analyzer total root length, total number of root tips, total root surface area and the average root diameter.

### Measurements of total chlorophyll (Chl) and carotenoids (Car) concentration

Leaf discs were harvested 13 days after transplanting and weighed and placed in 5 ml of N, N-dimethylformamide (N,N-DMF, Sigma Chemical Co.) in darkness for 48 hours at 4°*C*. The absorption of pigments were measured using a spectrophotometer (UV-2550 Shimadzu, Japan) at 647 nm, 664 nm and 480 nm respectively. Chl a, Chl b and Car concentrations were calculated as described by Wellburn [31].

### Measurement of Chl fluorescence F_v_/F_m_ ratio

The maximum photochemical efficiency of PSII was estimated in dark-adapted samples by the F_v_/F_m_ ratio. After 12 days of transplanting, F_v_/F_m_ ratio were measured during mid-photoperiod using the Plant Efficiency Analyser (Hansatech Instruments, UK) according to He et al. [32].

### Measurements of electron transport rate (ETR), photochemical quenching (qP) and non-photochemical quenching (NPQ)

After 11 days of transplanting, ETR, qP and NPQ were determined at 25°*C* in the laboratory. Prior to measurements, the youngest fully expanded leaves were pre-darkened for 15 min. By using the PAM Chl Fluorometer (Walz, Effeltrich, Germany), images of fluorescence emission were digitized within the camera and via a Firewire interface (400 megabits/s) (Firewire-1394, Austin TX, USA) to a personal computer for storage and analysis. Measurements and calculations of ETR, qP and NPQ were described previously [24].

### Measurements of photosynthetic CO_2_ assimilation rate (*A*), stomatal conductance (*g*_*s*_), internal CO_2_ concentration (*C*_*i*_) and transpiration (*T*_*r*_)

After 20 days of transplanting, fully expanded leaves were selected. After the plants were exposed to LED lighting for 4 to 5 hours, *A*, *g*_*s*_, *C*_*i*_, and *T*_*r*_ were measured and recorded simultaneously from intact leaves in the indoor farming room using the LI-COR Portable Photosynthetic System (LI-64000. Bioscience, USA) which has a LED light source that supplied PPFDs of 250 (similar to growth irradiance) and higher PPFDs of 1000 and 1500 μmol m^-2^ s^-1^. According our previous report [24], photosynthetic rate measured by oxygen evolution of *M*. *crystallinum* leaf discs did not saturate at a high light intensity of 1500 μmol m^-2^ s^-1^. The wavelength of the light source emitted ranged between 420 nm to 510 nm and 610 nm to 730 nm. The average ambient CO_2_ concentration was 430 ± 10 μmol mol^-1^, relative humidity was around 60% and leaf chamber temperature was set to prevailing ambient temperature of 25°*C*.

### Measurement of NO_3_^-^ concentration

Dried tissues of 0.01g were grounded with 5 ml deionised water, incubated at 37°C for 2 hours and centrifuged at 3500 rpm for 5 min. The mixture was filtered through a 0.45 um-pore-diameter membrane via a vacuum filter to remove sample turbidity. The flow injection analyser (Model Quickchem 800, Lachat Instruments Inc., Milwaukee, USA) was used to determine NO_3_^-^ concentration by catalytically reducing NO_3_^-^ to NO_2_^-^ by passing the sample through a copperized cadmium column. NO_2_^-^ was diazotized with sulphanilamide followed by coupling with N-(1-naphthyl) ethylenediamine dihydrochloride resulting in a water soluble dye with a magenta colour which was read at 520 nm.

### Measurement of total reduced nitrogen (TRN) content

Dried shoot tissues (0.05 g) were digested with a Kjeldahl tablet and 5 ml of concentrated sulphuric acid for 60 min at 350° C and the mixture was allowed to cool before TRN was determined by a Kjeltec 2300 analyzer (Foss Tecator AB, Höganäs, Sweden) through titration. The concentration of TRN was calculated as a unit of mg g^-1^ DW.

### Determination of total leaf soluble protein and Rubisco protein by SDS-PAGE

Fresh leaves of 1g were ground to fine powder with liquid nitrogen. A total of 6 ml of extraction buffer [100mM Bicine-KOH (pH 8.1), 20mM MgCl_2_, 2% PVP buffer] was added to the powder and mixed thoroughly. The mixture was centrifuged at 35,000 rpm for 30 min at 4°*C (*Beckman ultracentrifuge Optima XL-100K). After centrifugation, 2 ml of the supernatant was mixed with 8 ml of 80% cold acetone and centrifuged for 10 min at 4,000 rpm. The amount of total soluble protein was determined using the method of Lowry et al. [33]. Protein extract of was diluted (1:1 ratio) with solubilizing solution (20% glycerol, 0.02% bromophenolblue, 5% SDS, 0.125M Tris and 10% B-mercaptoethanol) before loading onto a pre-cast gradient gel (PROTEAN TGX precast gel, any KD, BIO-RAD, USA). Electrophoresis was performed under constant voltage. The gel was then stained in coomassie brilliant blue (0.2% coomassie brilliant blue in 10% acetic acid, 50% methanol) and destained with 7% acetic acid and 25% ethanol. Fluor Chem 8800 gel imaging system was used to analyse the resultant bands (S1 Fig) under visible light.

### Determination of total ascorbic acid (ASC) content

Ascorbic (ASC, reduced form) and dehydroascorbic (DHASC oxidized form) acids were assayed by the reduction of 2,6-dichlorophenolindophenol (DCPIP) according to Leipner et al., [34]. Fresh leaves of 0.5g were grounded to powder with liquid nitrogen, 1g of NaCl was added and extraction was carried out by adding 5ml of ice-cold 2% (w/v) metaphosphoric acid. The homogenate was centrifuged at 9,000 rpm for 30 min at 4°*C*. An aliquot of 0.3 ml was mixed with 0.2 ml 45% (w/v) K_2_HPO_4_ and 0.1 ml 0.1% (w/v) homocysteine to reduce DHASC to ASC and determine the total ASC pool (ASC + DHASC). After 15 min of incubation at 25°C, 1 ml of 2 M citrate–phosphate buffer (pH 2.3) and 1 ml 0.003% (w/v) DCPIP were added. The absorbance at 524 nm was immediately measured using a spectrophotometer (UV-2550 Shimadzu, Japan).

### Determination of total phenolic compounds

Fresh samples (0.5g) were ground in liquid nitrogen and 5 ml of 80% methanol [35]. The extracts were shaken at 200 rpm for 30 min, centrifuged at 3500 rpm for 20 min. The analysis of total phenolic compounds involved diluting 0.5 ml of supernatant with 0.5 ml of Folin-Ciocalteau reagent and 1 ml of 7.5% sodium bicarbonate solution and left for 20 min. The absorbances at 740 nm were recorded using a spectrophotometer (UV-2550 Shimadzu, Japan).

### Determination of soluble sugars

After 15 days of transplanting, leaf tissue was dried at 80°C for 4 days. For extraction, 10 mg of dried tissue was added to hot 80% ethanol (2 ml, heated to 65*°*C), agitated for 30 min, centrifuged for 5 min at 3500 rpm and supernatant was collected. The pellet was re-suspended in an additional 2 ml of hot 80% ethanol and the process was repeated twice. The concentration of free soluble sugar was determined colorimetrically at 490 nm using a spectrophotometer (UV-2550 Shimadzu, Japan) according to Dubois et al. [36].

### Determination of proline concentration

This assay was modified from the protocol by Bates et al. [37]. Frozen tissue of 0.5 g was grounded with 6 ml of 3% sulfosalicylic acid and centrifuged at 9000 rpm for 10 min at 4°*C*. The supernatant (1 ml) was mixed with equal volumes of acid-ninhydrin and acetic acid and the mixture was heated in a water bath at 95*°*C for an hour. The reaction was stopped by placing the mixture in ice. The reaction mixture was extracted with 2 ml of toluene, vortexed for 30 seconds. The absorbance was read at 520 nm using a spectrophotometer (UV-2550 Shimadzu, Japan).

### Determination of dietary minerals

Dried tissues of 0.2 g were microwave-digested in 4 ml of 65% nitric acid using UltraWAVE (Milestone, US). Digested samples were diluted with Milli-Q water to final volume of 25 ml. The data was obtained from conducting inductively coupled plasma optical emission Elmer, US) [38]. Concentrations of Ca, K, Mg and Fe were calculated from the data collected.

### Statistical analysis

To ensure that the variances across samples in groups that had different nutrient spraying frequencies are equal, Levene’s test was carried out. One-way analysis of variances (ANOVA) and Tukey’s multiple comparison tests were carried out to discriminate between the means of the different groups, where p>0.05 indicates that the means are significantly different. The statistical analysis was performed using SPSS statistics software.

## Results

### Shoot and root productivity, leaf growth and leaf water status

**Fig 1:**
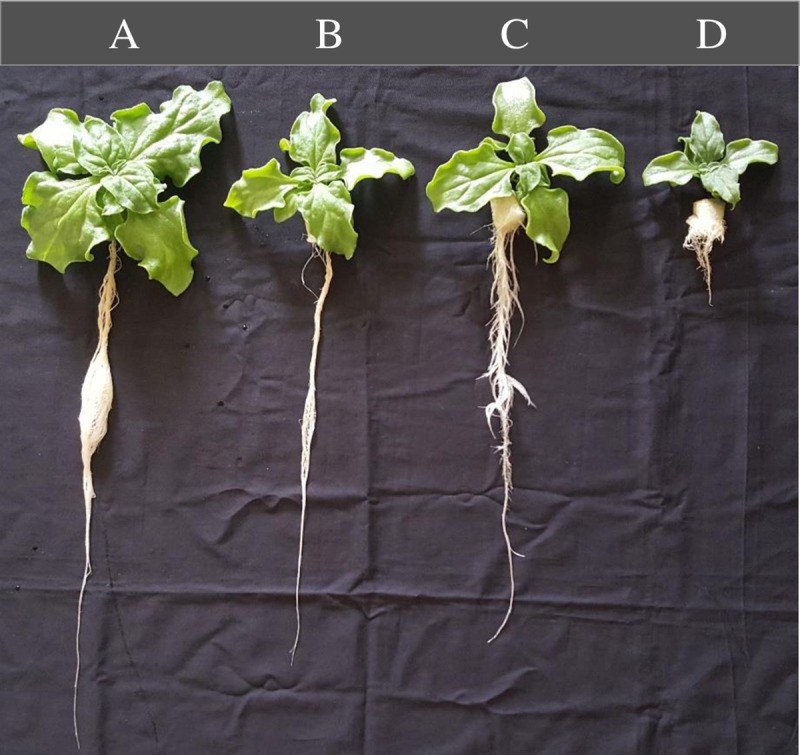
*M*. *crystallinum* grown under different nutrient spraying intervals for 15 days. A, B, C and D were 5, 30, 60 and 240 min nutrient spraying intervals, respectively.

**Fig 2:**
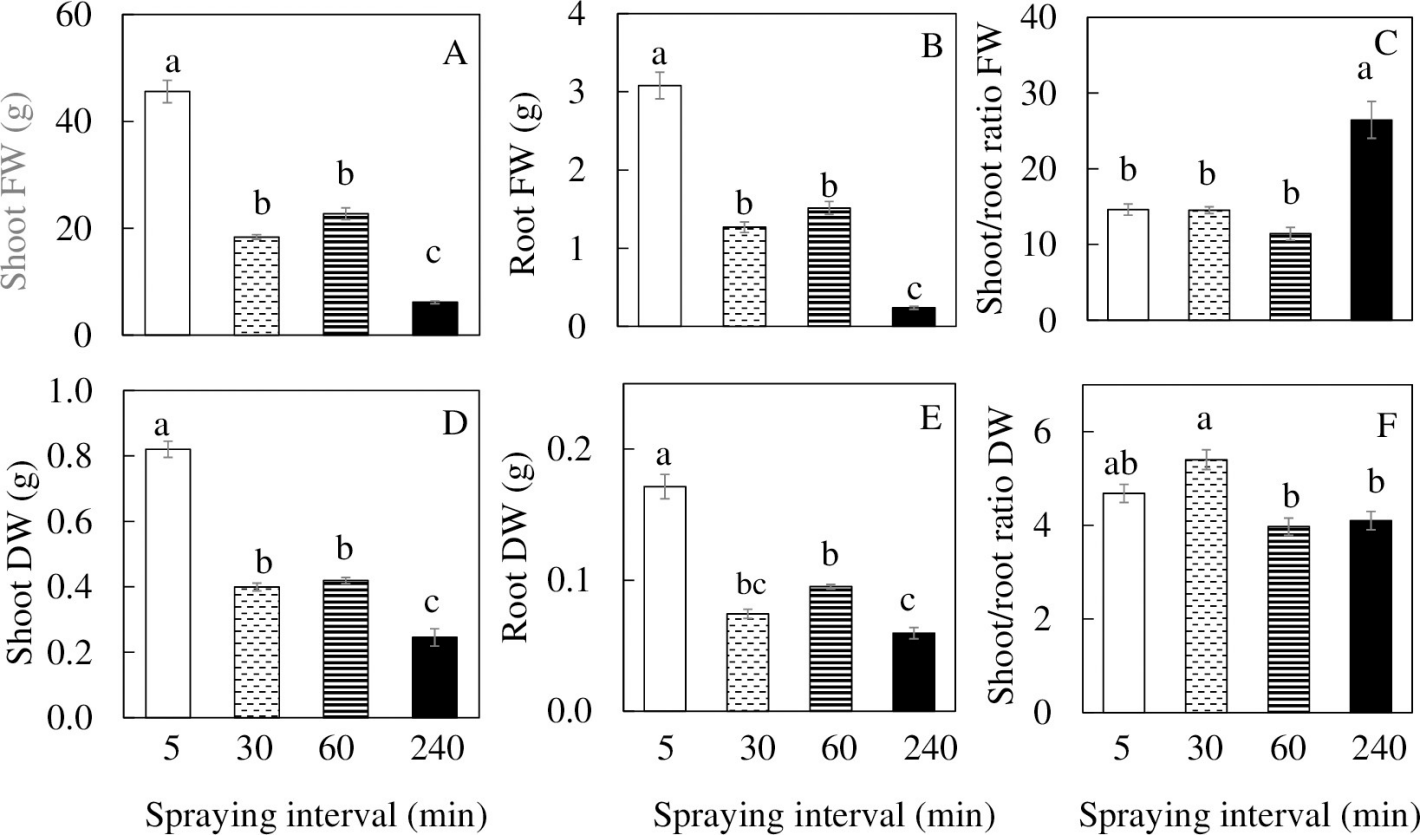
Shoot FW and DW (A, D), root FW and DW (B, E), shoot/root ratio FW and DW (C, F) of *M*. *crystallinum* grown under different nutrient spraying intervals for 15 days. Vertical bars represent the standard errors. Means with different letters are statistically different (P < 0.05; n = 4) as determined by Tukey's multiple comparison test.

**Fig 3:**
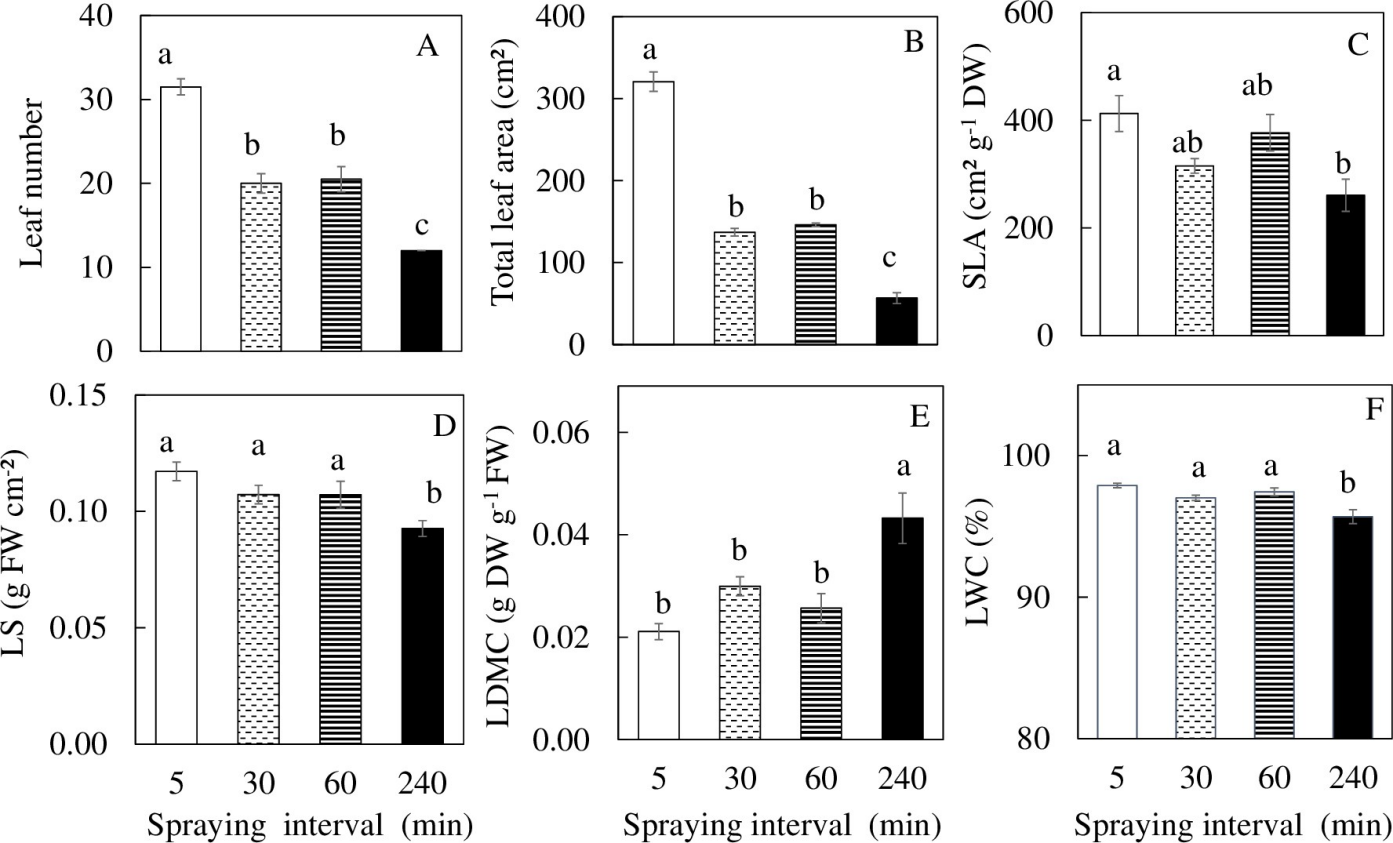
Leaf number (A), total leaf area (B), SLA (C), LS (D), LDMC (E) and LWC (F) of *M*. *crystallinum* grown under different nutrient spraying intervals for 15 days. Vertical bars represent the standard errors. Means with different letters are statistically different (P < 0.05; n = 4) as determined by Tukey's multiple comparison test.

Fig 1 shows a 15-day *M*. *crystallinum* plants with different nutrient spaying intervals. The shoot, and root FW (Fig 2A and 2B) and DW (Fig 2D and 2E) were significantly higher for plants grown under 5 min nutrient spraying interval than under all other spraying intervals. Plants grown under 30 and 60 min nutrient spraying intervals had similar values for shoot, root FW and DW. Plants grown under the 240 min nutrient spraying interval had significantly lower values (Fig 2A, 2B, 2D and 2E). The shoot/root ratio FW for the plants grown under 240 min nutrient spraying intervals was significantly higher, almost double in value than the other groups (Fig 2C). For the plants grown under 5 min nutrient spraying interval, the shoot/root ratio DW had no significant difference from the other groups. Among the other groups, the shoot/root ratio DW of plants grown under 30 min nutrient spraying intervals was significantly higher than those grown under 60 and 240 min nutrient spraying intervals (Fig 2F). The leaf number and total leaf area of plants grown under 5 min nutrient spraying interval were significantly higher than all other groups with no significant differences between plants grown under 30 and 60 min nutrient spraying intervals. The plants grown under 240 min nutrient spraying interval had significantly lower leaf number and total leaf area compared to other plants (Fig 3A and 3B). For SLA, only plants grown under 240 min nutrient spraying interval was significantly lower than under 5 min nutrient spraying intervals (Fig 3C). For plants grown under 240 min nutrient spraying intervals, the LS and LWC (Fig 3D and 3F) were significantly lower while LDMC (Fig 3E) were significantly higher compared to all other plants.

### Root morphology

**Fig 4:**
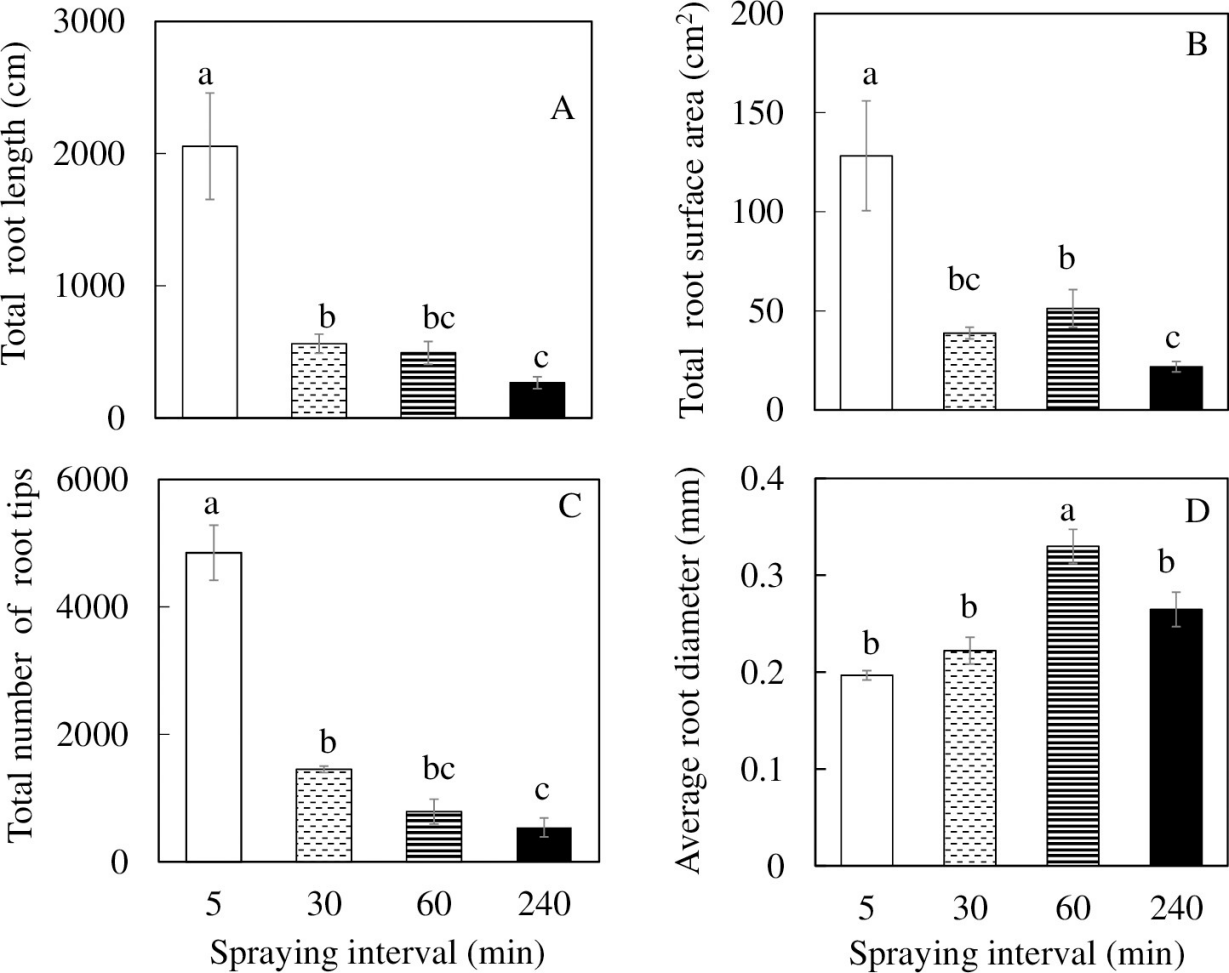
Total root length (A), total root surface area (B), total number of root tip (C), and average root diameter (D) of *M*. *crystallinum* grown under different nutrient spraying intervals for 9 days. Vertical bars represent the standard errors. Means with different letters are statistically different (P < 0.05; n = 4) as determined by Tukey's multiple comparison test.

The total root length, total root surface area and total number of root tips of plants grown under 5 min nutrient spraying interval were significantly higher than all other plants. The values of these parameters for plants grown under 30 and 60 min nutrient spraying intervals were similar (Fig 4A–4C). For the average root diameter, plants grown under the 60 min nutrient spraying interval was significantly higher than under 5, 30 and 240 min nutrient spraying intervals (Fig 4D).

### Photosynthetic pigments

**Fig 5:**
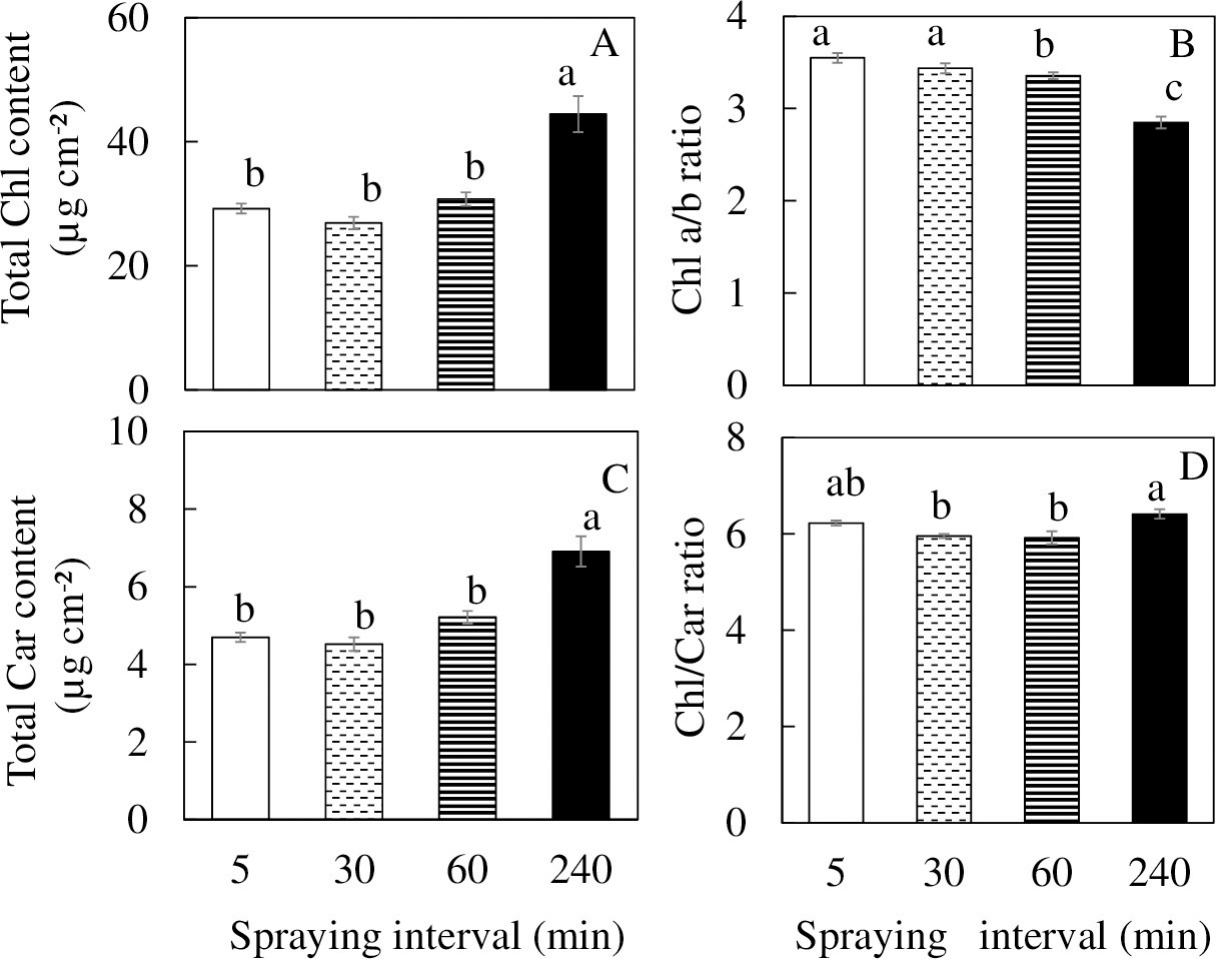
Total Chl content (A), Chl a/b ratio (B), total Car content (C), and Chl/Car ratio (D) of *M*. *crystallinum* grown under different nutrient spraying intervals for 12 days. Vertical bars represent the standard errors. Means with different letters are statistically different (P < 0.05; n = 4) as determined by Tukey's multiple comparison test.

For the plants grown under 240 min nutrient spraying interval, the total Chl and total Car contents were significantly higher than plants grown under other nutrient spraying intervals (Fig 5A and 5C). Chl a/b ratio of plants grown under 5 and 30 min nutrient spraying intervals were significantly higher than under 60 and 240 min spraying intervals. Grown under 240 min spraying interval, *M*. *crystallinum* had the lowest Chl a/b ratio (Fig 5B). For the Chl/Car ratio, only *M*. *crystallinum* grown under 240 min nutrient spraying interval was significantly higher than those grown under 60 min nutrient spraying interval (Fig 5D).

### F_v_/F_m_ ratio, ETR, qP and NPQ

**Fig 6:**
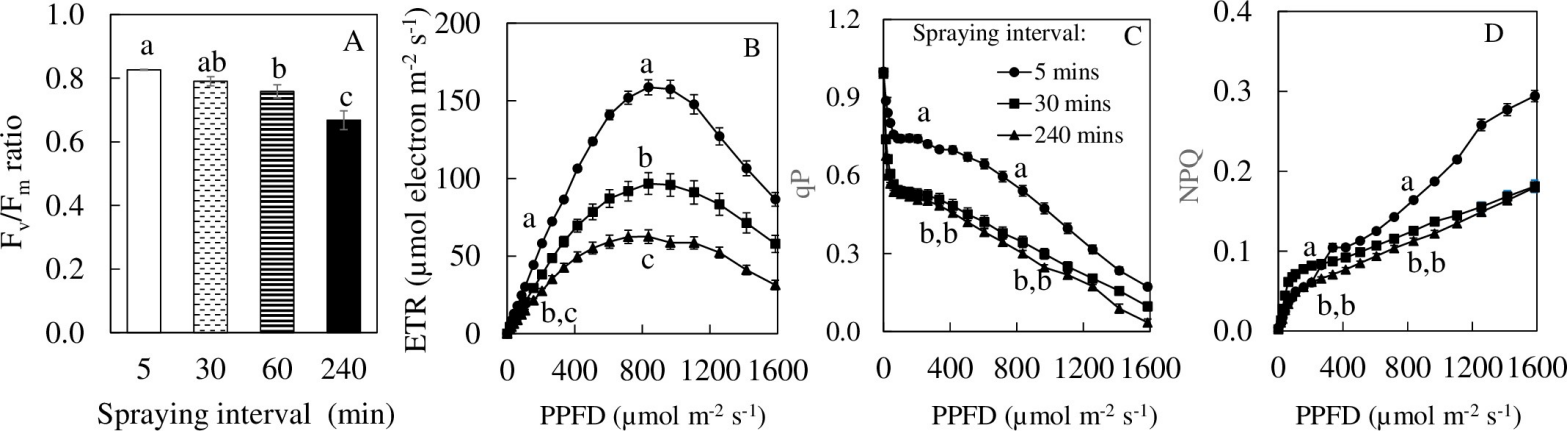
F_v_/F_m_ ratio (A), light response curves of ETR (B), qP (C) and NPQ (D) of *M*. *crystallinum* grown under different nutrient spraying intervals. Each point is the mean of 20 measurements of 4 different leaves from 4 different plants (n = 20). Vertical bars represent the standard errors. Means with different letters are statistically different (P < 0.05; n = 4) as determined by Tukey's multiple comparison test. Light response curves of ETR, qP and NPQ were similar for *M*. *crystallinum* grown under 30 and 60 min nutrient spraying intervals. Curves for 60 min in B, C, and D were remove for clearance.

*M*. *crystallinum* grown under 5 and 30 min nutrient spraying intervals had F_v_/F_m_ ratios above 0.8 and were significantly higher than plants grown under 240 min (Fig 6A). Plants grown under 60 and 240 min nutrient spraying intervals had F_v_/F_m_ ratios of less than 0.8, indicating that they were under stress. Under the growth irradiance of PPFD 250 μmol m^-2^ s^-1^ and the saturated light intensity of PPFD 836 μmol m^-2^ s^-1^, ETR of *M*. *crystallinum* grown under 5 min nutrient spraying interval being the highest followed by the plants grown under 30 and 240 min nutrient spraying intervals (Fig 6B). Under both the growth and saturated irradiances, qP of *M*. *crystallinum* from 5 min nutrient spraying interval were significantly higher than *M*. *crystallinum* from 30 and 240 min nutrient spraying intervals (Fig 6C). NPQ of *M*. *crystallinum* grown under 5 and 240 min nutrient spraying interval were significantly lower than those from 30 min nutrient spraying intervals at the growth irradiance of PPFD 250 μmol m^-2^ s^-1^. At the saturated light intensity of PPFD 836 μmol m^-2^ s^-1^, NPQ of *M*. *crystallinum* grown under 5 min nutrient spraying interval was significantly higher than those grown under 30 and 60 min nutrient spraying intervals (Fig 6D).

### *A*, *g*_*s*_, *C*_*i*_ and *T*_*r*_

**Fig 7:**
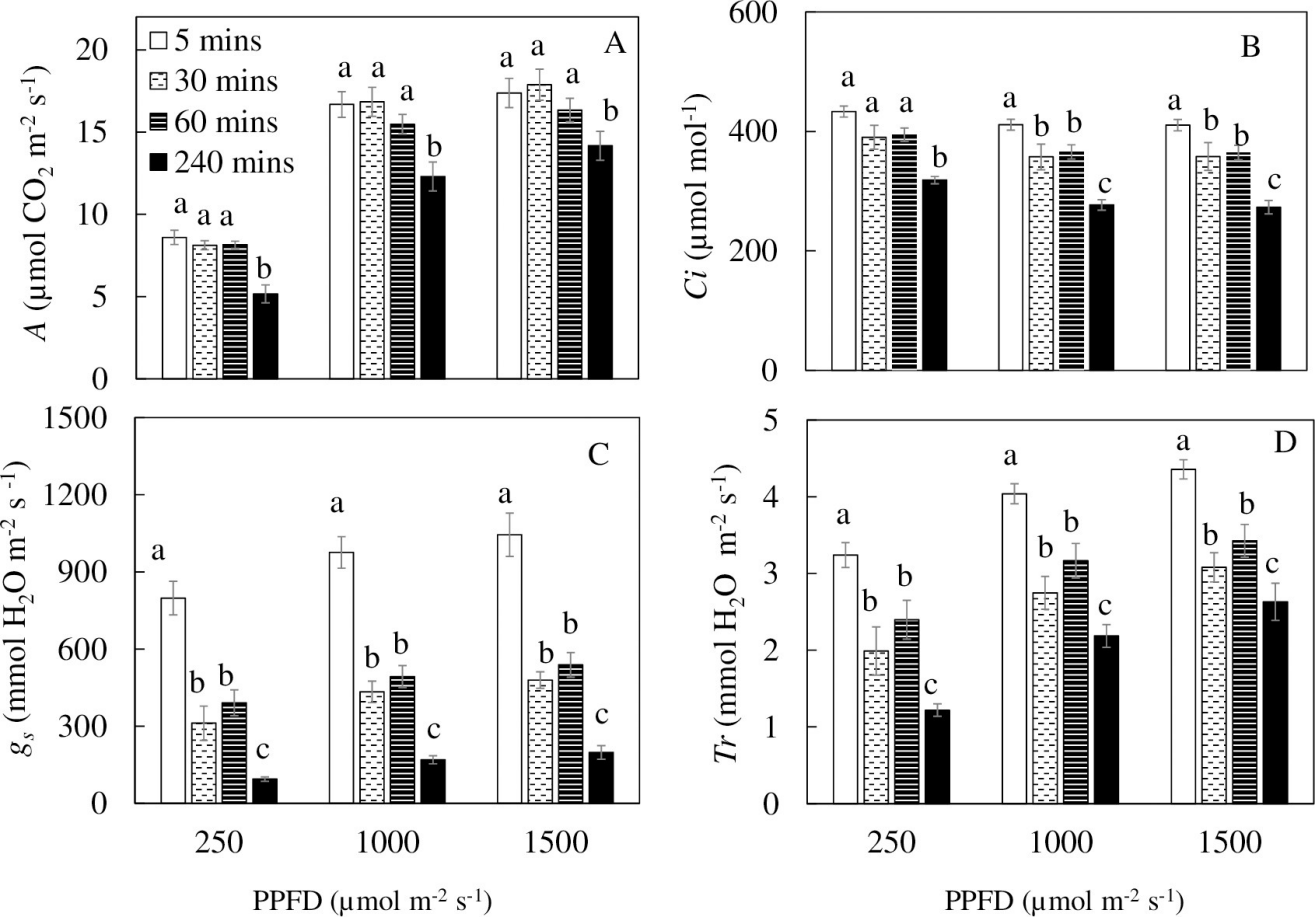
*A* (A); *C*_*i*_ (B); *g*_*s*_ (C) and *T*_*r*_ (D) of *M*. *crystallinum* grown under different nutrient spraying intervals for 20 days. The measurements were made under PPFDs of 250, 1000 and 1500 μmol photon m^-2^ s^-1^. Vertical bars represent the standard errors. Means with different letters are statistically different (p<0.05; n = 4) as determined by Tukey's multiple comparison test.

Measured under PPFD of 250 μmol m^-2^ s^-1^, which was close to the growth irradiance, and two other higher PPFDs of 1000 and 1500 μmol m^-2^ s^-1^, values of *A* from *M*. *crystallinum* plants grown under 240 min nutrient spraying interval were significantly lower compared to all other plants (Fig 7A). At a PPFD 250 μmol m^-2^ s^-1^, *C*_*i*_ of *M*. *crystallinum* grown under 240 min nutrient spraying interval were also significantly lower than those of other plants. At PPFDs of 1000 and 1500 μmol m^-2^ s^-1^, *C*_*i*_ values of *M*. *crystallinum* grown under 5 min nutrient spraying interval were significantly higher than those grown under 30 and 60 min nutrient spraying intervals (Fig 7B). *M*. *crystallinum* grown under 240 min nutrient spraying interval had the lowest *C*_*i*_ measured at both PPFDs 250, 1000 and 1500 μmol m^-2^ s^-1^. At all three PPFDs, *g*_*s*_, of *M*. *crystallinum* grown under 5 min nutrient spraying interval was significantly higher than all other plants. For instance, the *g*_*s*_ of plants under 5 min nutrient spraying interval was at least double of those grown under 30 and 60 min nutrient spraying intervals and at least five times greater than those grown under 240 min nutrient spraying interval (Fig 7C). For the values of *Tr*, *M*. *crystallinum* grown under 5 min nutrient spraying interval were significantly higher than those grown under other spraying intervals measured at all PPFDs (Fig 7D).

### NO_3_^-^, leaf TRN, leaf total soluble protein and Rubisco protein

**Fig 8:**
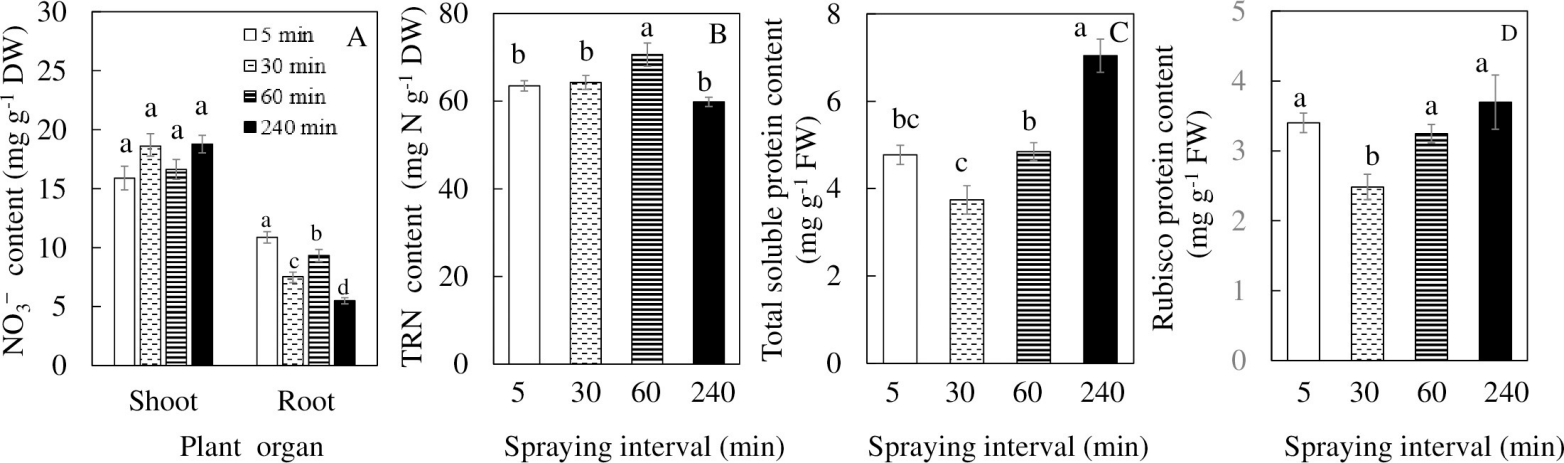
Total NO_3_^-^ (A), TRN (B), total soluble protein (C) and Rubisco protein contents (D) of *M*. *crystallinum* grown under different nutrient spraying intervals for 15 days. Vertical bars represent the standard errors. Means with different letters are statistically different (P < 0.05; n = 4) as determined by Tukey's multiple comparison test.

There were no significant differences in shoot NO_3_^-^ content among the different nutrient spraying intervals but there were significant differences among the root NO_3_^-^ content with *M*. *crystallinum* grown under 5 min nutrient spraying interval having the highest root NO_3_^-^ content followed by 60, 30 and 240 min nutrient spraying intervals (Fig 8A). There were no significant differences in TRN among *M*. *crystallinum* grown in 5, 30 and 240 min nutrient spraying intervals, only *M*. *crystallinum* grown under 60 min nutrient spraying interval had a significantly higher TRN than those of the rest plants (Fig 8B). Total soluble protein was significantly higher in *M*. *crystallinum* grown under 240 min nutrient spraying interval compared to those of all other plants (Fig 8C). There were no significant difference in Rubisco content among the *M*. *crystallinum* grown in 5, 60 and 240 min nutrient spraying intervals except for a lower Rubisco content in the plants grown under 30 min nutrient spraying intervals (Fig 8D).

### Phytochemicals and inorganic dietary minerals

**Fig 9:**
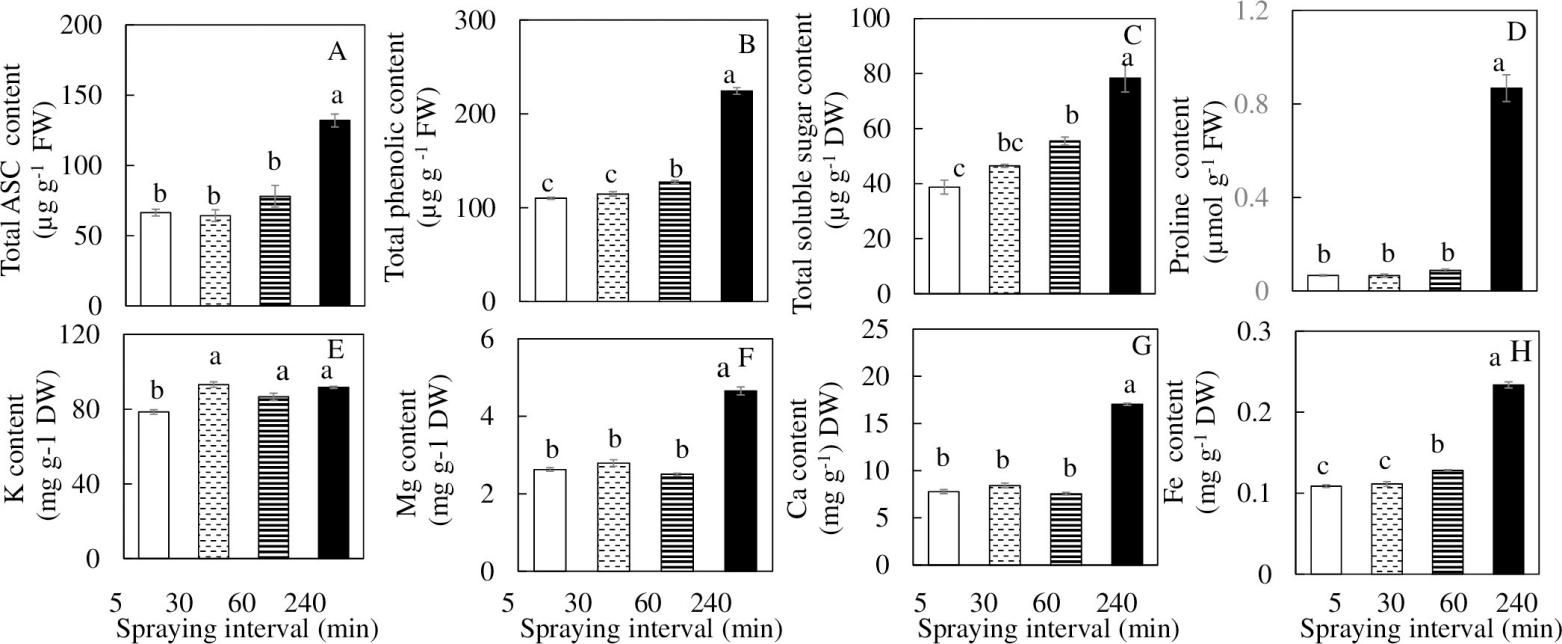
Total ASC (A), total phenolic compounds (B), total soluble sugar (C), proline (D), K (E), Mg (F), Ca (G) and Fe (H) contents of *M*. *crystallinum* grown under different nutrient spraying intervals for 15 days. Vertical bars represent the standard errors. Means with different letters are statistically different (P < 0.05; n = 4) as determined by Tukey's multiple comparison test.

Similar trends were observed in total ASC, total phenolic, soluble sugar and proline contents. The contents of these phytochemicals were significantly higher in *M*. *crystallinum* grown under 240 min nutrient spraying interval compared to other intervals (Fig 9A–9D). The contents of total ASC and total phenolic were almost two-fold higher in the plants grown under 240 min nutrient spraying intervals than the other spraying intervals (Fig 9A and 9B). Proline content in *M*. *crystallinum* grown under 240 min nutrient spraying interval was at least 8-fold greater than the other plants grown under different spraying intervals (Fig 9D). The contents of K in *M*. *crystallinum* grown under 5 min nutrient spraying intervals was significantly lower than those grown under other nutrient spraying intervals (Fig 9E). There were similar trends observed in the Mg, Ca and Fe contents where *M*. *crystallinum* grown under 240 min nutrient spraying interval had significantly higher content of these dietary minerals than under 5, 30 and 60 min nutrient spraying intervals (Fig 9F–9H).

## Discussion

Our previous study showed that *M*. *crystallinum* plants performed C_3_ photosynthesis with high growth rate and had a very low CAM acidity when supplied with adequate water using hydroponic cultivation under LED lighting [24]. In the present study, all plants were grown aeroponically with our own design systems [39]. Aeroponics is the cultivation of plants with their root systems suspended in the air while a nutrient solution is misted to maintain a constant film of nutrient and moisture on the root surface. *M*. *crystallinum* grown under 5 min nutrient spraying interval had significantly much higher shoot and root FW and DW (Figs 1, 2A, 2B, 2D and 2E); greater leaf number, and larger total leaf area (Fig 3A–3C) compared to the other longer nutrient spraying intervals. These findings indicate that there is reduced total leaf area for photosynthesis which ultimately affects productivity when *M*. *crystallinum* was supplied with less nutrient solution with extended spaying intervals. Drought stress, a deficiency of water in an environment has been observed to cause reduced leaf growth and leaf areas in many other plants like poplar [40] and soybean [41]. Would *M*. *crystallinum* grown under longer nutrient spraying intervals (simulation of drought stress) experience varying degrees of water deficit stress, the lack of water in plant tissues causing damage to plants, and mineral deficiency? For this facultative species, would CAM be induced by drought as reported in *Cissus trifoliata* [42], *Talinum triangulare* [43] and *Clusia* species [44]?

A common adverse effect of drought stress is typically the most limiting factor for plant growth, biomass accumulation, and productivity [45]. Furthermore, studies have shown that drought stress in an important limiting factor during the initial phase of plant growth as it affects elongation and expansion growth of cells [46]. The reduction in total leaf area of *M*. *crystallinum* grown under 30, 60 and 240 min nutrient spraying intervals (Fig 3A) might have resulted from inhibition of new leaf development with less number of shoot branches (S2 Fig) and decrease of individual leaf expansion [47]. The results of the present study showed that *M*. *crystallinum* with less nutrient solution spraying to roots resulted in lower shoot (Fig 2A and 2D) and root productivity (Fig 2B and 2E). When grown in soil, water uptake may be limited by the amount of roots. Enhanced root growth and/or partitioned high amount of dry matter to root is of crucial importance for productivity under drought stress [48]. However, in the present study, compared to plants grown under 5 min nutrient spraying interval, both root FW and DW were 2- to 4-fold lower for plants grown under 30, 60 and 240 min nutrient spraying intervals (Fig 2B and 2E). The higher shoot/root ratio FW of plants with 240 min spraying interval (Fig 2C) was mainly due to its greater reduction of root FW (Fig 2B). This was not seen in shoot/root ratio DW (Fig 2F) due to the lower LWC (Fig 3F). Similar to plants grown in the soil, the amount of water and nutrient available to an aeroponically grown plant is also determined by the root surface area, the amount of branching and the distances to which the root extend horizontally and vertically [49–51]. When plants are exposed to drought stress, the morphological characteristics of root change in correspondence with the water and nutrient uptake [52]. In the present study, decreases in total root length, total surface area and total number of root tips were observed across *M*. *crystallinum* grown under increasing nutrient spraying intervals (Fig 4A–4C). The average root diameter of *M*. *crystallinum* grown under 60 min nutrient spraying interval was the highest (Fig 4D). This may be a result of the *M*. *crystallinum* plants were trying to cope with the reduced water supply. These findings were similar to our previous work on lettuce plants grown under hot ambient temperature with root thickening compared to those grown under cool root-zone temperature [49–51]. This result contradicts the proposition that there was a decrease in root diameter under drought conditions to increase acquisition of water [53]. However, there is a reduction in root diameter seen in *M*. *crystallinum* grown under the 240 min nutrient spraying interval, probably due to the reduced amount of carbon fixed (Fig 7A, to be discussed later in more detail).

Inhibition of root growth and alternation of root morphology of aeroponically grown plants affect their capabilities in acquiring water, hence affecting the plant water status. This is in spite the fact that they were continuously supplied with nutrient solution [49–51, 54–56]. Leaf succulence valuated by FW per leaf area and LWC were lower for *M*. *crystallinum* grown under the 240 min nutrient spraying interval than under other three nutrient spraying intervals (Fig 3A and 3B). Leaf succulence is a common feature of CAM plants suggesting its role for stored water [57]. In the present study, lower leaf succulence may be due to the less external water supply when plants were subjected the longest duration of 240 min nutrient spraying intervals. Higher LDMC (Fig 3E) for *M*. *crystallinum* grown under the 240 min nutrient spraying interval probably resulted from its lower LWC (Fig 3F) not higher DW accumulation. Thus, lower SLA (cm^2^ g^-1^ DW) in these plants (Fig 3C) indicate that they had thicker leaves not greater LDMC as SLA is a compound trait, a function of LDMC and leaf thickness described by Hodgson et al. [58]. With more nutrient spraying to its roots, *M*. *crystallinum* generally showed greater SLA and lower LDMC.

Both Chl and Car play important roles in harvesting light and protect plants from drought stress [59]. However, results of this study showed otherwise. Instead of decreases in their contents, the total Chl and Car contents were significantly higher for *M*. *crystallinum* grown under 240 min nutrient spraying interval (Fig 5A and 5C). This could be due to reduced total leaf area (Fig 3B) of the whole plants grown under 240 min nutrient spraying interval and thus higher total Chl and Car concentrations on an area basis. In another word, lower pigment concentrations in *M*. *crystallinum* grown under shorter nutrient spraying intervals could have been due to a dilution effect. In study with chickpea, Rahbariani et al. [60] also found the increases of Chl and Car content that may be related to a decrease in leaf area. They viewed that as a defensive strategy to reduce detrimental effects under drought stress. Lower Chl a/b ratios in *M*. *crystallinum* grown were observed under 60 and 240 min nutrient spraying intervals (Fig 5B). However, higher Chl/Car ratio for *M*. *crystallinum* grown under 240 min nutrient spraying interval (Fig 5D) merits our further study.

Grown under 5 and 30 min nutrient spraying intervals, F_v_/F_m_ ratios of *M*. *crystallinum* were close or above 0.8 while plants grown under 60 and 240 min had their F_v_/F_m_ ratio below 0.8 (Fig 6A), which may indicate reduced maximal quantum yield of PS II, resulting from drought stress [61]. However, some researchers pointed out that the F_v_/F_m_ ratio may be suitable for drought sensitive species but not for drought insensitive species [62,63]. Li et al. [64] reported that a severe drought episode during the peak vegetative growth stage of maize plants resulted in decreases in Chl content, F_v_*/*F_m_ ratio, qP but increases in NPQ. In the present study, with the increase of nutrient spray intervals especially at 240 min, qP, NPQ and ETR of *M*. *crystallinum* decreased at a growth irradiance of PPFD 250 μmol m^-2^ s^-1^ above the growth irradiance (Fig 6B–6D). These findings indicate that drought stress resulted in not only a decrease in utilization of photochemical energy but also a reduction in the ability of *M*. *crystallinum* to dissipate excessive light energy as heat energy. There are a number of studies showed that drought stress resulted in a decline in NPQ [65]. It was also reported that NPQ increases under moderate drought stress but decreases under severe drought stress [65]. Although NPQ harmlessly dissipate excess energy, an enhanced NPQ may increase energy cost for CO_2_ fixation at a given light intensity [66]. Decreases of NPQ in *M*. *crystallinum* grown under longer duration of nutrient spraying interval may be due to the limitation of newly fix carbon resulting from reduction in photosynthetic gas exchange (Fig 7A).

Reduction in photosynthetic gas exchange was observed through the significantly reduced values of *A*, *Ci*, *g*_*s*_ and *Tr* of *M*. *crystallinum* grown under 240 min nutrient spraying intervals (Fig 7) at both the growth irradiance of PPFD 250 μmol m^-2^ s^-1^ and higher light intensities of PPFD 1000 and 1500 μmol m^-2^ s^-1^. Low *Ci* (Fig 7B) observed in *M*. *crystallinum* grown under 240 min nutrient spraying interval was most likely a result of the lowered *g*_*s*_ (Fig 7C) to prevent or reduce the loss of water as water availability is scarce [67], which supported by lower Tr (Fig 7D). As a result, *Ci* became limited and thus reduced *A* (Fig 7A). It was reported that CAM may be induced by drought for facultative species [42–44]. Although *M*. *crystallinum* is a facultative CAM plant, in the present study, switch between C_3_ photosynthesis and CAM did not occur under drought conditions as there was no CAM activity observed. CAM activity was measured by diurnal changes in titratable acidity as a proxy for large diurnal fluctuation in malate produced by night-time fixation of CO_2_ and daytime decarboxylation of malate [68]. However, in the present study, CAM activity was undetectable. Under induced drought conditions (i.e. 240 min spraying interval), *M*. *crystallinum* performed C_3_ photosynthesis, instead of CAM to cope in those harsh conditions, suggest that this plant may engage other mechanism(s). For instance, osmotic adjustment through the accumulation of different solutes such as soluble sugars (Fig 9C) and proline (Fig 9D) in the cytosol to lower osmotic potential and maintain cell turgor, is one of the strategies to cope with drought stress [14,15].

Based on results of leaf succulence (Fig 3D) and LWC (Fig 3F), water deficit had occurred in *M*. *crystallinum* grown under 30, 60 and 240 min nutrient spraying intervals compared to those grown under 5 min nutrient spraying intervals. We have previously reported that poor root development of aeroponically grown plants resulted in water deficit [39]. In the present study, lower root NO_3_^-^ content was also obtained from *M*. *crystallinum* grown under longer nutrient spraying intervals compared to those grown under 5 min spraying interval (Fig 8A). Would longer nutrient spraying intervals resulting in water deficit also cause nutrient deficiency? For soil grown plants, most of dissolved mineral nutrient transport from the soil solution to the roots mainly depends on the water potential gradient between soil and roots. Thus, under drought stress conditions, water deficit and consequent nutrient deficiency would occur [69]. Previously we have reported that poor root development of aeroponically grown plants resulted in not only water deficit but also negative impact on the mineral uptake and assimilation under hot root-zone temperature in the tropical greenhouse [39]. In the present study, longer nutrient spraying interval means less water and nutrition delivered to the roots. However, when nutrient solution was spraying to the roots which were hanging in the air, the roots rapidly absorbed both water and nutrient and transported them to the aerial part regardless of spraying intervals. The longer spraying intervals with less water supplied to the root resulted in inhibition of new leaf development (Fig 3A and 3B) with less number of shoot branches (S2 Fig). Due to their slow growth and small size, long nutrient spraying intervals did not seem to cause nutrient deficiency in *M*. *crystallinum*. This postulation was supported by the fact that nitrogen deficiency did not seemed to occur in the shoots as there were no significant differences in shoot NO_3_^-^ content among the different plants grown under different nutrient spraying intervals (Fig 8A). Furthermore, all plants had adequate shoot TRN which was greater than 2% (Fig 8B). According to Epstein [70], adequate tissue level of N that may be required by plants is around 1.5%. This may explain the high Chl content in *M*. *crystallinum* grown under the 240 min nutrient spraying interval as these plants have a much smaller area, and since NO_3_^-^ content was not reduced, high Chl was possibly a way for the plant to store N [71]. Another possible mean where N was stored within the small *M*. *crystallinum* would be in total soluble and Rubisco [72]. This was supported by the fact that *M*. *crystallinum* grown 240 min nutrient spraying interval had highest total soluble protein compared to other plants (Fig 8C). Despite having a much lower shoot FW and DW compared to plants grown at 5 min nutrient spraying frequency, *M*. *crystallinum* grown at 240 min nutrient spraying interval had similar amount of Rubisco The amount of Rubisco is controlled by the rate of synthesis and degradation even under stressful conditions [73, 74]. Webber et al. [75] reported that Rubisco is relatively stable with a half‐life of several days under drought stress. Although stomatal close is the most evident response to drought stress, which resulted in resulted in low *g*_*s*_ (Fig 7C) and *Ci* (Fig 7B), with high N content, *M*. *crystallinum* grown under 240 min nutrient spraying interval could afford to continue Rubisco synthesis. Rubisco is an inefficient enzyme and it is often the limiting step in photosynthesis [76]. High amount of Rubisco protein for *M*. *crystallinum* grown at 240 min nutrient spraying interval rule out the possibility of Rubisco loss that resulted in reduction of *A* (Fig 7A) under drought stress. However, Rubisco activity may have been affected by drought stress [77], which merits our further study. It was reported that Rubisco activation decreases in cotton under drought stress due to their lower *g*_*s*_ resulting in warmer leaf temperature [77].

*M*. *crystallinum* is able to grow in salinity and drought conditions, which generate oxidative stresses [11,78]. The ability of plants to overcome harmful environment is attribute to the important antioxidant biosynthesis, mainly ascorbic acids, ASC [78] and phenolics [19]. Salt and drought tolerance halophyte plants is often associated with accumulation of soluble sugars [11,46] and proline [14,15] for osmotic adjustment. In the present study, *M*. *crystallinum* grown under 240 min nutrient spraying interval had significantly higher ASC, total phenolics, total soluble sugar and proline concentration than those grown under other nutrient spraying intervals (Fig 9A–9D). The higher concentrations of these phytochemicals could be a result of the smaller area. The lower levels of phytochemicals observed *M*. *crystallinum* grown under 5, 30 and 60 min nutrient spraying intervals are a result of dilution as the plants have a much higher shoot FW (Fig 2A) and larger total leaf area (Fig 3C) than those grown under the 240 min nutrient spraying interval. High total ASC concentration in *M*. *crystallinum* grown under 240 min nutrient spraying interval shows correspondence to the low *Ci* (Fig 7B) and *g*_*s*_ (Fig 7C) to protect plants from photooxidation [74]. Lower F_v_/F_m_ ratio (Fig 7A), ETR (Fig 7B) and qp (Fig 7C) imply that photooxidation would likely to take place. By having higher concentration of ASC, it functions as an antioxidant by removing more H_2_O_2_ formed by O_2_ photoreduction in PSI during Mehler reaction [79] to prevent photoxidative stress. However, the highest Chl content (Fig 5A) obtained from *M*. *crystallinum* grown under 240 min nutrient spraying interval indicate no photooxidation had occurred in these plants. Both genetics and environment affect the level of phenolic compounds [80, 81]. The moderate drought stress treatment (50% field capacity) increased total phenolic compound of *Achillea* species [80]. Studying with artichoke, Nouraei et al. [81] found that most phenolic acids increased under drought stress, which could be a biochemical response to cope with oxidative stress. It has also been reported that plant accumulates soluble sugars in thyme [45] under drought conditions. High proline levels in *M*. *crytallinum* grown under the 240 min nutrient spraying interval proves that the plant is undergoing drought stress [80,81]. Nouraei, et al. [81] reported that proline content was significantly increased in leaves and in heads of artichoke, suggesting that such amino acid could be involved in the osmoregulation and antioxidant defense mechanisms, contributing to balanced ROS in both leaves and heads. Gharibi et al. [80] also observed that the proline content of the leaves increased in *Achillea* species under drought stress.

Mineral concentration was not adversely affected in *M*. *crystallinum* grown under longer nutrient spraying intervals. In fact, similarly to the high phytochemicals for plants grown under 240 min nutrient spraying interval, high mineral concentrations could due to minerals being concentrated within a small plant. With economic advancements in developed countries like Singapore, people are increasingly becoming more particular about the quality of food. *M*. *crystallinum* grown under 240 min nutrient spraying interval had a much lower yield in terms of shoot FW than the other plants grown under higher nutrient spraying frequencies. However, phytochemicals and the inorganic dietary minerals except for K are in fact 2 to 8 folds higher compared to other plants grown under shorter nutrient spraying intervals. As such, nutritional content of aeroponically grown *M*. *crystallinum* was enhanced when it experienced drought stress. However, CAM regulation and photosynthetic performance of *M*. *crystallinum* by drought for enhancing nutritional quality of *M*. *crystallinum* could be dependent on the intensity of growth irradiance which merits our further study.

## Conclusion

Although its CAM photosynthesis was not induced, the facultative *M*. *crystallinum* grown aeroponically had experienced water deficit stress under longer nutrient solution spraying intervals than under the shortest interval of 5 min. Plants grown under 240 min spraying interval utilized lesser light energy and had reduced *A*, *g*_*s*_, *C*_*i*_ and *T*_*r*_ measured at both growth and saturated irradiances. Mineral deficiency was not observed in plants grown under longer nutrient spraying intervals regardless of their much smaller root size. Instead, all plants had similar values of shoot NO_3_^-^, TRN, total soluble protein and Rubisco concentrations. For plants grown under 240 min spraying interval, they had significantly higher phytochemicals and dietary minerals than the other plants due to its smaller size. In order to produce high nutritional ice plants it is recommendable to grow *M*. *crystallinum* with longer nutrient spraying interval using indoor aeroponic farming systems by increasing sowing density to compensate lower shoot productivity. This strategy will enhanced nutritional quality at low production cost through water and nutrient management.

## Supporting information

S1 Fig SDS-PAGE of *M*. *crystallinum* leaf soluble protein. Soluble protein was isolated from leaf supernatant fractions of *M*. *crystallinum* grown under different nutrient spraying intervals for 15 days. The lane M contains Precission Plus Protein Standards (BIO-RAD).
(TIF)

S2 Fig Shoot branches of *M*. *crystallinum* grown under different nutrient spraying intervals for 15 days. Vertical bars represent the standard errors. Means with different letters are statistically different (P < 0.05; n = 4) as determined by Tukey's multiple comparison test.
(TIF)
