# Supplementary figures and images for: Drought does not induce crassulacean acid metabolism (CAM) but regulates photosynthesis and enhances nutritional quality of Mesembryanthemum crystallinum
Source: PLoS One. 2020 Mar 6;15(3):e0229897. doi: 10.1371/journal.pone.0229897 (PMC7059918; doi:10.1371/journal.pone.0229897)

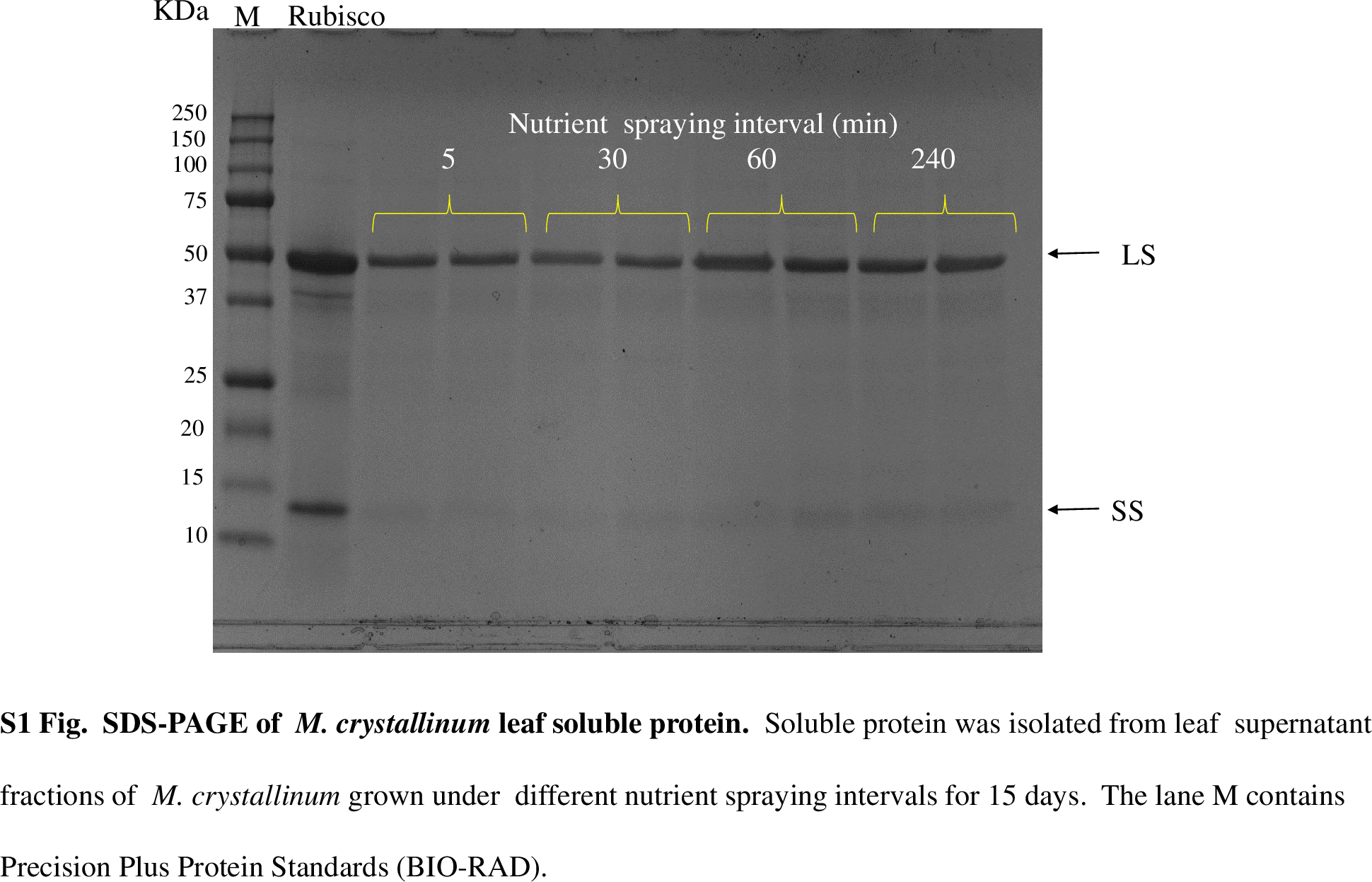

Supplement: S1 Fig — Soluble protein was isolated from leaf supernatant fractions of M. crystallinum grown under different nutrient spraying intervals for 15 days. The lane M contains Precission Plus Protein Standards (BIO-RAD). (TIF) [file pone.0229897.s001.tif]

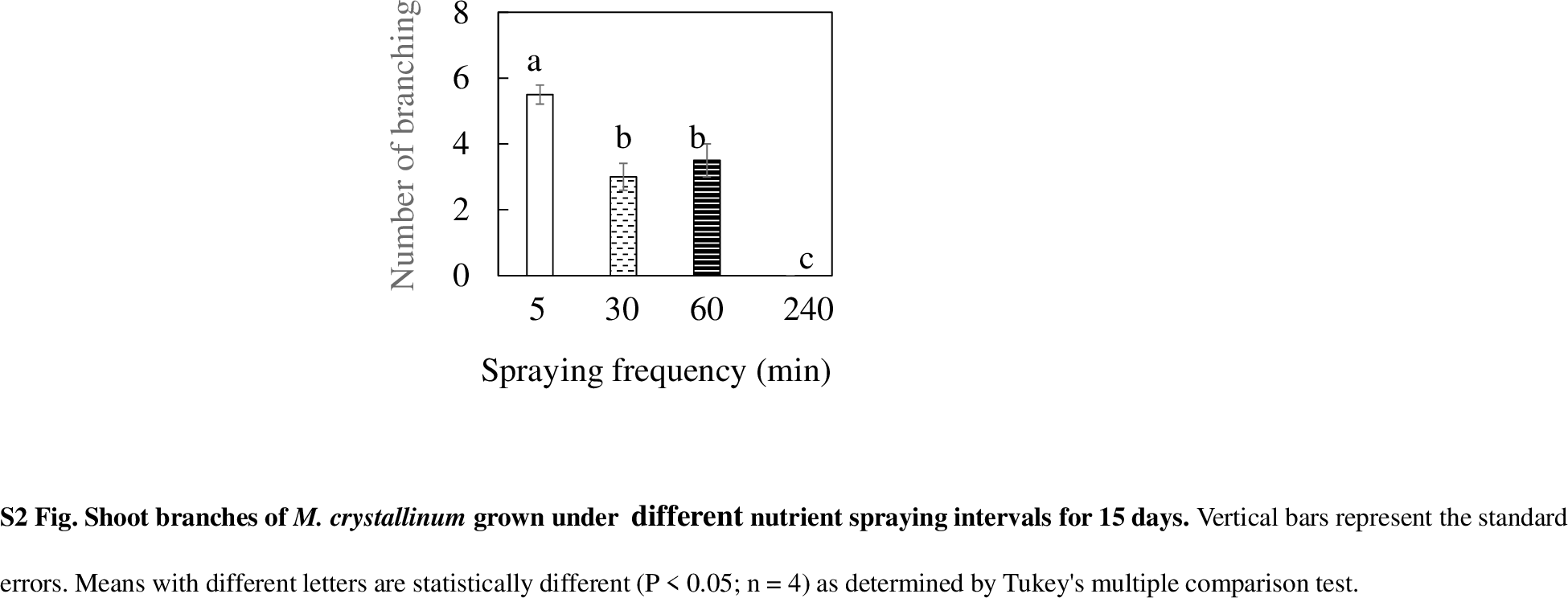

Supplement: S2 Fig — Vertical bars represent the standard errors. Means with different letters are statistically different (P < 0.05; n = 4) as determined by Tukey's multiple comparison test. (TIF) [file pone.0229897.s002.tif]
